# Differences in Health Care, Family, and Community Factors Associated with Mental, Behavioral, and Developmental Disorders Among Children Aged 2–8 Years in Rural and Urban Areas — United States, 2011–2012

**DOI:** 10.15585/mmwr.ss6608a1

**Published:** 2017-03-17

**Authors:** Lara R. Robinson, Joseph R. Holbrook, Rebecca H. Bitsko, Sophie A. Hartwig, Jennifer W. Kaminski, Reem M. Ghandour, Georgina Peacock, Akilah Heggs, Coleen A. Boyle

**Affiliations:** 1 Division of Human Development and Disability, National Center on Birth Defects and Developmental Disabilities, CDC, Atlanta, Georgia; 2 Oak Ridge Institute for Science and Education, CDC Research Participation Programs, Oak Ridge, Tennessee; 3 Office of Epidemiology and Research, Maternal and Child Health Bureau, Health Resources and Services Administration, Rockville, Maryland; 4 Office of the Director, National Center on Birth Defects and Developmental Disabilities, CDC, Atlanta, Georgia

## Abstract

**Problem/Condition:**

Mental, behavioral, and developmental disorders (MBDDs) begin in early childhood and often affect lifelong health and well-being. Persons who live in rural areas report more health-related disparities than those in urban areas, including poorer health, more health risk behaviors, and less access to health resources.

**Reporting Period:**

2011–2012.

**Description of System:**

The National Survey of Children’s Health (NSCH) is a cross-sectional, random-digit–dial telephone survey of parents or guardians that collects information on noninstitutionalized children aged <18 years in the United States. Interviews included indicators of health and well-being, health care access, and family and community characteristics. Using data from the 2011–2012 NSCH, this report examines variations in health care, family, and community factors among children aged 2–8 years with and without MBDDs in rural and urban settings. Restricting the data to U.S. children aged 2–8 years with valid responses for child age and sex, each MBDD, and zip code resulted in an analytic sample of 34,535 children; MBDD diagnosis was determined by parent report and was not validated with health care providers or medical records.

**Results:**

A higher percentage of all children in small rural and large rural areas compared with all children in urban areas had parents who reported experiencing financial difficulties (i.e., difficulties meeting basic needs such as food and housing). Children in all rural areas more often lacked amenities and lived in a neighborhood in poor condition. However, a lower percentage of children in small rural and isolated areas had parents who reported living in an unsafe neighborhood, and children in isolated areas less often lived in a neighborhood lacking social support, less often lacked a medical home, and less often had a parent with fair or poor mental health.

Across rural subtypes, approximately one in six young children had a parent-reported MBDD diagnosis. A higher prevalence was found among children in small rural areas (18.6%) than in urban areas (15.2%). In urban and the majority of rural subtypes, children with an MBDD more often lacked a medical home, had a parent with poor mental health, lived in families with financial difficulties, and lived in a neighborhood lacking physical and social resources than children without an MBDD within each of those community types. Only in urban areas did a higher percentage of children with MBDDs lack health insurance than children without MBDDs. After adjusting for race/ethnicity and poverty among children with MBDDs, those in rural areas more often had a parent with poor mental health and lived in resource-low neighborhoods than those in urban areas.

**Interpretation:**

Certain health care, family, and community disparities were more often reported among children with MBDDS than among children without MBDDs in rural and urban areas.

**Public Health Action:**

Collaboration involving health care, family, and community services and systems can be used to address fragmented services and supports for children with MBDDs, regardless of whether they live in urban or rural areas. However, addressing differences in health care, family, and community factors and leveraging community strengths among children who live in rural areas present opportunities to promote health among children in rural communities.

## Introduction

Mental health is a critical component of physical health and development. The onset of mental, behavioral, and developmental disorders (MBDDs) often occurs in childhood. Nationally representative data suggest that 15% of U.S. children aged 2–8 years (i.e., early childhood, as defined by Healthy People 2020 [HP2020]) (*1*) have a parent-reported MBDD diagnosis (*2*). Treating these conditions early is important; an HP2020 objective sets a national target for 76% of all children with mental health problems to receive treatment (*1*). Factors associated with having a parent-reported MBDD diagnosis in early childhood include inadequate insurance coverage, lacking a medical home (patient-centered, coordinated primary care model), fair or poor parental mental health, financial difficulties (i.e., “very hard to get by on your family’s income—hard to cover the basics like food or housing”), employment difficulties because of child care issues, living in a neighborhood lacking social support (i.e., neighbors who “help each other out,” “watch out for each other’s children,” and can be “count[ed] on” and “trusted to help my child”), and living in a neighborhood with limited amenities (i.e., no sidewalks, parks or playgrounds, recreation or community centers, or libraries) or in poor condition (i.e., with litter or garbage on the street or sidewalk, poorly kept housing, or vandalism (*2*). Understanding how these factors are associated with mental health among young children in different types of communities might help those who are developing prevention and intervention programs.

Persons who live in rural communities (compared with those in urban communities) often have health-related disparities, including worse health, more health risk behaviors, and less access to resources (*3*). Indicators of poor mental health among adults (e.g., serious mental illness among men, major depressive episodes among men and women, and recent serious psychological distress among women) have been found to be higher in large rural counties than in small rural, suburban, and urban counties (*3*). Most studies examining children’s mental health in rural and urban areas indicate comparable rates of mental disorders in the two types of areas (*4*,*5*). However, mental disorders might be underreported in rural areas (*6*). For example, in an analysis of the Hawaii public health system, children living in the most rural areas (small rural towns and isolated rural areas combined) had more substantial mental health needs than children in suburban areas at the time mental health treatment was initiated (*7*).

A 2005 Health Resources and Services Administration report described availability, accessibility, and acceptability as a framework to understand the key barriers that affect rural behavioral health (*8*); behavioral health includes the services and programs that prevent, diagnose, and treat symptoms of mental and neurodevelopmental disorders. The availability and quality of specialized behavioral health services and providers often are insufficient to serve children in rural communities (*9*). For example, 61.6% of areas with shortages of mental health professionals are in rural or partially rural areas (*10*). Differences in access to behavioral health care might be reflected in the type of care children receive. A study of 2002–2008 Medical Expenditure Panel Survey data indicated higher rates of psychopharmacological treatment compared with counseling services for children aged 5–17 years across community settings; in addition, compared with children in urban areas, children in rural areas had significantly higher rates of any prescriptions for mental disorders (*5*).

Behavioral health care in rural communities also can be affected by social acceptability factors such as stigma, cultural beliefs, and values unique to the rural community and community subgroups. Stigma and a lack of anonymity of behavioral health treatment in rural communities can contribute to delays in seeking care and underuse of care (*6*,*11*). Specifically, black youths in rural areas are half as likely as white youths in rural areas to use specialized mental health treatment; overall, among all racial/ethnic groups combined, parent reports of the effects of MBDDs on the family (i.e., economically, socially, and psychologically) and having public health insurance were positively associated with specialty mental health use (*12*).

Accessibility factors, such as lack of knowledge of behavioral health needs and treatment options, inadequate financing, limited transportation, and social isolation also can create behavioral health service barriers for youths in rural areas (*9*,*11*). Among parents of children with special health care needs (inclusive of MBDDs), those living in rural areas are more likely to report unmet health care needs caused by transportation and financial difficulties than those in urban areas (*13*). Recruiting and retaining specialized behavioral health providers can be challenging because of these barriers (*9*).

A 2015 White House initiative highlighted the need to strengthen the quality of, access to, and collaboration within early childhood learning programs, parenting support programs, health care, and economic support programs to address rural childhood poverty (*14*). This initiative also underscores the complexity of understanding the relationship between rurality and poverty. Suburban areas have had the lowest rates of persons who live below the poverty threshold and many of the most positive health outcomes, whereas the smallest, most isolated areas with the highest rates of poverty have reported the poorest health outcomes (*3*). The negative effects of childhood poverty on health and development are well documented (*14*,*15*). For example, parent reports of child height and weight from the 2011–2012 National Survey of Children’s Health (NSCH) indicated that more children aged 10–17 years in rural areas were overweight or obese than those in urban areas. In addition, both in urban and rural areas, lower income households were significantly more likely to have an overweight or obese child than households with higher incomes within those areas (*16*). Demographic and family factors (e.g., low maternal education, poverty, having public insurance coverage, and mental health impairment) have accounted for the increased likelihood of attention-deficit/hyperactivity disorder (ADHD) among children in rural areas compared with those in urban areas (*5*). Although substantial research indicates that living in neighborhoods with high poverty is associated with behavioral problems among young children (*15*), research that clarifies which specific neighborhood factors might be associated with mental health among children in rural communities is lacking.

The collective research suggests that sociodemographic, health care, and community factors are associated with MBDDs in children both in rural and urban settings. The prevalence of children’s MBDDs in rural areas might be confounded by some of these factors; therefore, examining the variables separately by area (i.e., urban vs. rural) is important. This report expands previous analyses of MBDDs and sociodemographic, health care, family, and community factors among U.S. children aged 2–8 years (*2*) by examining differences in these factors among children with and without MBDDs according to whether they live in in urban, large rural, small rural, or isolated areas. This report is intended for public health officials, clinicians, policymakers, and researchers who would like to understand and address factors associated with MBDDs among children in rural areas. Findings from this report might help different types of communities focus their mental health prevention and intervention efforts for young children while also helping achieve the HP 2020 objective that 76% of children with mental health problems receive treatment (*1*).

## Methods

CDC analyzed data from the 2011–2012 NSCH to examine differences in sociodemographic, health care, family, and community factors among children aged 2–8 years with and without MBDDs living in urban, large rural, small rural, and isolated areas. NSCH is a cross-sectional, random-digit–dial telephone survey of parents and guardians that collects information on noninstitutionalized children aged <18 years in the United States. Interviews included indicators of health and well-being, health care access, and family and community characteristics (*17*) (Table 1). For each identified household with children, parents and guardians responded to questions about one randomly selected child in the home. MBDD diagnosis was determined by parent report and was not validated with health care providers or medical records. Urban and rural designations were determined using a census tract–based classification system and work commuting information. 

**TABLE 1:** Questions and methods for the National Survey of Children’s Health related to mental, behavioral, and developmental disorders; rurality; and health care, family, and community factors — United States, 2011–2012

Variable	Questions and methods
MBDDs	Parent responded yes to at least one question: “Has a doctor or other health care provider ever told you that [child] had [specified MBDD]?” Specified MBDDs included ADHD, depression, anxiety problems, behavioral or conduct problems such as oppositional defiant disorder or conduct disorder, Tourette syndrome, autism spectrum disorder, learning disability, intellectual disability, developmental delay, or speech or other language problems.
Urban or rural residence	Urban and rural designations were determined using the four-category classification of the 2006 RUCAs, a census tract–based classification system.* Urban areas (RUCA codes 1.0, 1.1, 2.0, 2.1, 3.0, 4.1, 5.1, 7.1, 8.1, and 10.1) include metropolitan areas and surrounding towns from which commuters flow to an urban area; large rural areas (RUCA codes 4.0, 4.2, 5.0, 5.2, 6.0, and 6.1) include large towns (micropolitan areas) with populations of 10,000–49,999 and their surrounding areas; small rural areas (RUCA codes 7.0, 7.2, 7.3, 7.4, 8.0, 8.2, 8.3, 8.4, 9.0, 9.1, and 9.2) include small towns with populations of 2,500–9,999 and their surrounding areas; isolated areas (RUCA codes 10.0, 10.2, 10.3, 10.4, 10.5, and 10.6) are not near towns with a population of ≥2,500.
**Health care **	
Inadequate insurance	Parent responded “no” to at least one of five survey items included in four variables: 1) whether the child has current health insurance coverage; 2) whether the child had gaps in coverage in the past 12 months; 3) whether the coverage is sufficient to meet the child’s needs; 4a) whether the family pays out-of-pocket expenses, 4b) and if yes, whether these expenses are usually or always reasonable; and 5) whether insurance allows the child to see needed health care providers.
No medical home	This variable was assessed through 19 survey items coded into five variables and based on the parent reporting the child not having at least one of the following components of a medical home: having a personal doctor or nurse, having a usual place of care, receiving family-centered care and care coordination, and for children who need them, getting needed referrals.
**Family **	
At least one parent with fair or poor mental health	Parent responded “fair” or “poor” (compared with “excellent,” “very good,” or “good”) to one of two questions: “In general, what is the status of [child name]’s [mother’s/your] mental and emotional health?” and “In general, what is the status of [child name]’s [father’s/your] mental and emotional health?”
Financial difficulties	Parent responded “very often” or “somewhat often” (compared with “rarely” or “never”) when asked “Since [the child] was born, how often has it been very hard to get by on your family’s income, for example, was it hard to cover the basics like food or housing?”
**Community **	
Neighborhood with limited amenities	Parent responded “no” to at least one of the following statements: “Please tell me if the following places and things are available to children in your neighborhood, even if [the child] does not actually use them”: 1) sidewalks or walking paths; 2) a park or playground area; 3) a recreation center, community center, or boys’ or girls’ club; 4) a library or bookmobile.
Neighborhood in poor condition	Parent responded “yes” to any of the following three questions: “In your neighborhood, is there litter or garbage on the street or sidewalk? How about poorly kept or rundown housing? How about vandalism such as broken windows or graffiti?”
Neighborhood with little social support	Parents responded they “definitely agree,” “somewhat agree,” “somewhat disagree,” or “definitely disagree” to each of four statements about their neighborhood or community: “People in this neighborhood help each other out; we watch out for each other’s children in this neighborhood; there are people I can count on in this neighborhood; if my child were outside playing and got hurt or scared, there are adults nearby who I trust to help my child.” Responses were scored 1–4 (ranging from “definitely agree” through “definitely disagree”), and an average score was calculated; averages ≥2.25^†^ indicated a lack of social support.
Neighborhood unsafe	Parent reported “never” or “sometimes” (compared with “usually” or “always”) to the question, “How often do you feel [the child] is safe in your community or neighborhood?”

**Abbreviations:** ADHD = attention-deficit hyperactivity disorder; MBDD = mental, behavioral, and developmental disorder; RUCA = rural-urban commuting area.

* **Source:** US Department of Health and Human Services, Health Resources and Services Administration. The health and well-being of children in rural areas: a portrait of the nation 2011–2012. Rockville, MD: US Department of Health and Human Services; 2015. https://mchb.hrsa.gov/nsch/2011-12/rural-health/pdf/rh_2015_book.pdf

^†^ **Source:** Data Resource Center for Child and Adolescent Health, Child and Adolescent Health Measurement Initiative, Maternal and Child Health Bureau. 2011–2012 National Survey of Children’s Health. Child health indicator and subgroups. SAS codebook, Version 1.0. Baltimore, MD: Child and Adolescent Health Measurement Initiative; 2013. http://www.childhealthdata.org/docs/nsch-docs/sas-codebook_-2011-2012-nsch-v1_05-10-13.pdf

For the 2011–2012 NSCH, the interview completion rates (i.e., the percentage of households that completed interviews among all eligible households that were contacted) were 54.1% for the landline sample and 41.2% for the cell phone sample. The overall response rate among all eligible households, accounting for households that were not successfully contacted, was 23.0% (*17*). NSCH attempts to minimize nonresponse bias by incorporating nonresponse adjustments in the development of the sampling weights. Among the 50 U.S. states and the District of Columbia, a total of 847,881 households were screened for age-eligible children. Within these households, 187,422 reported age-eligible children living or staying in the household. A total of 95,677 interviews were completed (*17*).

Sociodemographic variables included the child’s sex, age, race/ethnicity, 200% of the federal poverty level (FPL) determined by income and family size (e.g., $44,700 for a family of four in 2011), highest education of the respondent or another adult in the household, and primary household language (English or other). The FPL variable in the NSCH public use file included data from multiple imputation for the 9.3% of the sample for which household income was missing. Differences in sociodemographic, health care, family, and community factors were assessed among children with and without MBDDs in urban and rural areas.

Restricting the data to U.S. children aged 2–8 years with valid responses for child age and sex, each MBDD, and zip code (from which rural-urban commuting areas were determined) (*18*) resulted in an analytic sample of 34,535 children. Data were weighted to account for unequal probability of household and child selection and for nonresponse. Statistical software was used to calculate weighted prevalence estimates and prevalence ratios with 95% confidence intervals (CIs) and to account for the complex sampling design. Adjusted prevalence ratios with 95% CIs were calculated using weighted logistic regression models adjusting for poverty (<200% FPL or ≥200% FPL) and race/ethnicity (non–Hispanic white or other). Estimates based on small sample sizes were suppressed for confidentiality; therefore, three variables included in the previous study (*2*) were not included here (i.e., child lacks preventive medical care, parent lacks emotional support, and parent reports child care problems). Statistical significance was determined using a ≤0.05 threshold for the p value associated with each prevalence ratio.

## Results

Sociodemographic factors varied both within and among residential categories (Table 2). A higher prevalence of children in rural areas (large rural, small rural, and isolated areas) than children in urban areas were non-Hispanic white (and were less often non-Hispanic black or Hispanic), lived in a poor or near-poor household (i.e., <200% of the FPL), lived in a household where the highest adult educational level was a high school education or less, and spoke English as their primary language.

**TABLE 2:** Demographic, health care, family, and community factors among children aged 2–8 years in urban, large rural, small rural, and isolated areas — National Survey of Children’s Health, United States, 2011–2012

Variable	Urban*	Large rural*		Small rural*		Isolated*	
	% (95% CI)^†^	% (95% CI)^†^	PR^§^ (95% CI)	% (95% CI)^†^	PR^§^ (95% CI)	% (95% CI)^†^	PR^§^ (95% CI)
**Overall (row %)**	**82.4 (81.6–83.1)**	**9.0 (8.4–9.6)**	**—**	**4.9 (4.5–5.3)**	**—**	**3.7 (3.4–4.1)**	**—**
**Demographic **							
Race/Ethnicity							
White, non-Hispanic	47.4 (46.1–48.7)	62.8 (59.0–66.4)	1.3 (1.2–1.4)^¶^	66.0 (61.5–70.2)	1.4 (1.3–1.5)^¶^	71.0 (66.0–75.5)	1.5 (1.4–1.6)^¶^
Black, non-Hispanic	14.0 (13.2–15.0)	9.0 (7.2–11.2)	0.6 (0.5–0.8)^¶^	7.3 (5.7–9.4)	0.5 (0.4–0.7)^¶^	5.7 (3.7–8.5)	0.4 (0.3–0.6)^¶^
Hispanic	27.3 (25.9–28.7)	18.9 (15.4–22.9)	0.7 (0.6–0.8)^¶^	18.1 (14.1–22.8)	0.7 (0.5–0.8)^¶^	13.0 (9.3–18.0)	0.5 (0.3–0.7)^¶^
Other, non-Hispanic	11.2 (10.4–12.1)	9.4 (7.8–11.3)	0.8 (0.7–1.0)	8.6 (7.0–10.6)	0.8 (0.6–1.0)**	10.4 (8.2–13.1)	0.9 (0.7–1.2)
<200% federal poverty level	43.7 (42.3–45.0)	58.5 (55.2–61.8)	1.3 (1.3–1.4)^¶^	61.0 (56.4–65.4)	1.4 (1.3–1.5)^¶^	52.8 (48.0–57.6)	1.2 (1.1–1.3)^¶^
No more than high school education in household	48.7 (47.4–50.1)	57.2 (53.9–60.5)	1.2 (1.1–1.3)^¶^	55.4 (50.9–59.7)	1.2 (1.1–1.3)^¶^	56.7 (52.0–61.4)	1.3 (1.1–1.4)^¶^
English as primary household language	80.5 (79.2–81.7)	90.4 (87.0–92.9)	1.1 (1.1–1.2)^¶^	89.7 (86.0–92.5)	1.1 (1.1–1.2)^¶^	91.3 (87.7–94.0)	1.1 (1.1–1.2)^¶^
**Health care **							
Inadequate insurance	21.5 (20.4–22.6)	20.7 (17.9–23.8)	1.0 (0.8–1.1)	19.7 (16.4–23.5)	0.9 (0.8–1.1)	21.1 (17.5–25.1)	1.0 (0.8–1.2)
No medical home	44.6 (43.2–46.0)	44.3 (40.8–47.8)	1.0 (0.9–1.1)	41.6 (37.5–45.9)	0.9 (0.8–1.0)	36.3 (31.8–41.0)	0.8 (0.7–0.9)^¶^
**Family **							
At least one parent with fair or poor mental health	11.2 (10.2–12.3)	13.6 (11.1–16.7)	1.2 (1.0–1.5)	13.3 (9.8–17.7)	1.2 (0.9–1.6)	8.2 (6.1–10.8)	0.7 (0.5–1.0)**
Financial difficulties	25.1 (23.9–26.3)	30.6 (27.5–34.0)	1.2 (1.1–1.4)^¶^	29.8 (26.2–33.7)	1.2 (1.0–1.4)**	27.0 (23.1–31.2)	1.1 (0.9–1.3)
**Community **							
Neighborhood with limited amenities	39.4 (38.0–40.7)	52.8 (49.3–56.4)	1.3 (1.2–1.4)^¶^	59.8 (55.6–64.0)	1.5 (1.4–1.6)^¶^	68.6 (63.8–73.0)	1.7 (1.6–1.9)^¶^
Neighborhood in poor condition	27.6 (26.4–28.9)	33.8 (30.4–37.3)	1.2 (1.1–1.4)^¶^	33.1 (29.5–37.0)	1.2 (1.1–1.4)^¶^	34.1 (29.8–38.8)	1.2 (1.1–1.4)^¶^
Neighborhood with little social support	20.0 (18.9–21.2)	18.7 (16.0–21.7)	0.9 (0.8–1.1)	18.1 (14.8–22.1)	0.9 (0.7–1.1)	9.1 (7.1–11.5)	0.5 (0.4–0.6)^¶^
Neighborhood unsafe	15.3 (14.3–16.4)	13.2 (10.4–16.6)	0.9 (0.7–1.1)	11.6 (9.0–14.7)	0.8 (0.6–1.0)**	6.1 (4.2–8.8)	0.4 (0.3–0.6)^¶^
**Any MBDD**	15.2 (14.3–16.1)	16.6 (14.5–19.0)	1.1 (0.9–1.3)	18.6 (15.6–22.0)	1.2 (1.0–1.5)**	15.8 (12.9–19.1)	1.0 (0.8–1.3)

**Abbreviations:** CI = confidence interval; MBDD = mental, behavioral, and developmental disorder; PR = prevalence ratio; RUCA = rural-urban commuting area.

* Urban and rural designations were determined using the four-category classification of the 2006 RUCAs, a census tract–based classification system. Urban areas (RUCA codes 1.0, 1.1, 2.0, 2.1, 3.0, 4.1, 5.1, 7.1, 8.1, and 10.1) include metropolitan areas and surrounding towns from which commuters flow to an urban area; large rural areas (RUCA codes 4.0, 4.2, 5.0, 5.2, 6.0, and 6.1) include large towns (micropolitan areas) with populations of 10,000–49,999 and their surrounding areas; small rural areas (RUCA codes 7.0, 7.2, 7.3, 7.4, 8.0, 8.2, 8.3, 8.4, 9.0, 9.1, and 9.2) include small towns with populations of 2,500–9,999 and their surrounding areas; isolated areas (RUCA codes 10.0, 10.2, 10.3, 10.4, 10.5, and 10.6) are not near towns with a population of ≥2,500 (**Source:** US Department of Health and Human Services, Health Resources and Services Administration. The health and well-being of children in rural areas: a portrait of the nation 2011–2012. Rockville, MD: US Department of Health and Human Services; 2015. https://mchb.hrsa.gov/nsch/2011-12/rural-health/pdf/rh_2015_book.pdf).

^†^ Percentages are weighted in the table.

^§^ Urban is referent group.

^¶^ Prevalence ratio significant at p<0.01.

** Prevalence ratio significant at p<0.05.

Three health care and family factors differed among rural areas (large rural, small rural, and isolated areas) (Table 2). A lower percentage of children in isolated areas than children in urban areas were reported to lack a medical home and have a parent with fair or poor mental health; children in large rural and small rural areas more often lived in families with financial difficulties than children in urban areas. Several community factors differed among residential categories. Children in all rural areas more often lacked amenities such as parks, recreation centers, sidewalks, and libraries in their neighborhood than children in urban areas and more often lived in a neighborhood in poor condition (i.e., with garbage, vandalism, or housing in poor condition). Children in small rural and isolated areas lived in an unsafe neighborhood less often than children in urban areas, and children in isolated areas less often lived in a neighborhood lacking social support. Prevalence of MBDDs among U.S. children aged 2–8 years was higher in small rural areas (18.6%) than in urban areas (15.2%); prevalence of MBDDs in large rural and isolated areas did not differ from urban areas.

**FIGURE 1:**
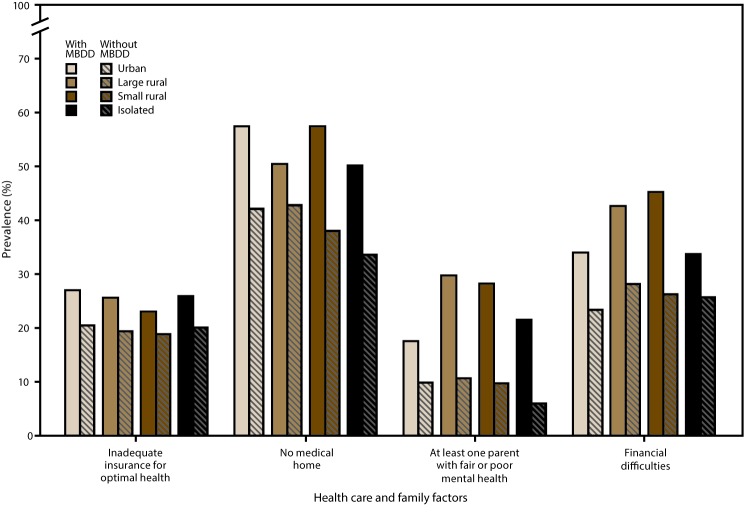
Prevalence of selected health care and family factors* among children aged 2–8 years with and without mental, behavioral, and developmental disorders^†^ in urban and rural areas^§^ — National Survey of Children’s Health, United States, 2011–2012 **Abbreviations:** MBDD = mental, behavioral, or developmental disorder; RUCA = rural-urban commuting area. * **Inadequate insurance:** Based on a negative response to one of five variables included in the following questions: 1) whether the child has current health insurance coverage; 2) whether the child had gaps in coverage in the past 12 months, 3) whether the coverage is sufficient to meet the child’s needs; 4a) whether the family pays out-of-pocket expenses, and if yes, 4b) whether these expenses are usually or always reasonable; and 5) whether insurance allows the child to see needed health care providers. **No medical home:** To have a medical home, children must have a personal doctor or nurse, usual source of care, and family-centered care; children needing referrals or care coordination must also have those criteria met. **Parent with fair or poor mental health:** Based on responses of “fair" or "poor” (i.e., compared with "excellent," "very good," or "good") to questions about maternal and paternal mental health. Maternal question: “In general, what is the status of [child name]’s [mother’s/your] mental and emotional health?” Paternal question: “In general, what is the status of [child name]’s [father’s/your] mental and emotional health?” **Financial difficulties:** Based on responses of “very often” or “somewhat often” (compared with "rarely" or "never") to “Since [the child] was born, how often has it been very hard to get by on your family’s income, for example, it was hard to cover the basics like food or housing?” ^†^ Significant differences in the prevalence of health care and family factors were found between children with and without MBDDs in certain urban and rural areas. **Inadequate insurance:** urban areas; **no medical home:** urban, small rural, and isolated areas; **parent with fair or poor mental health:** urban, large rural, small rural, and isolated areas; **financial difficulties:** urban, large rural, and small rural areas. ^§^ Urban and rural designations were determined using the four-category classification of the 2006 RUCAs, a census tract–based classification system. Urban areas (RUCA codes 1.0, 1.1, 2.0, 2.1, 3.0, 4.1, 5.1, 7.1, 8.1, and 10.1) include metropolitan areas and surrounding towns from which commuters flow to an urban area; large rural areas (RUCA codes 4.0, 4.2, 5.0, 5.2, 6.0, and 6.1) include large towns (micropolitan areas) with populations of 10,000–49,999 and their surrounding areas; small rural areas (RUCA codes 7.0, 7.2, 7.3, 7.4, 8.0, 8.2, 8.3, 8.4, 9.0, 9.1, and 9.2) include small towns with populations of 2,500–9,999 and their surrounding areas; isolated areas (RUCA codes 10.0, 10.2, 10.3, 10.4, 10.5, and 10.6) are not near towns with a population of ≥2,500. (**Source:** US Department of Health and Human Services, Health Resources and Services Administration. The health and well-being of children in rural areas: a portrait of the nation 2011–2012. Rockville, MD: US Department of Health and Human Services; 2015. https://mchb.hrsa.gov/nsch/2011-12/rural-health/pdf/rh_2015_book.pdf)

**FIGURE 2:**
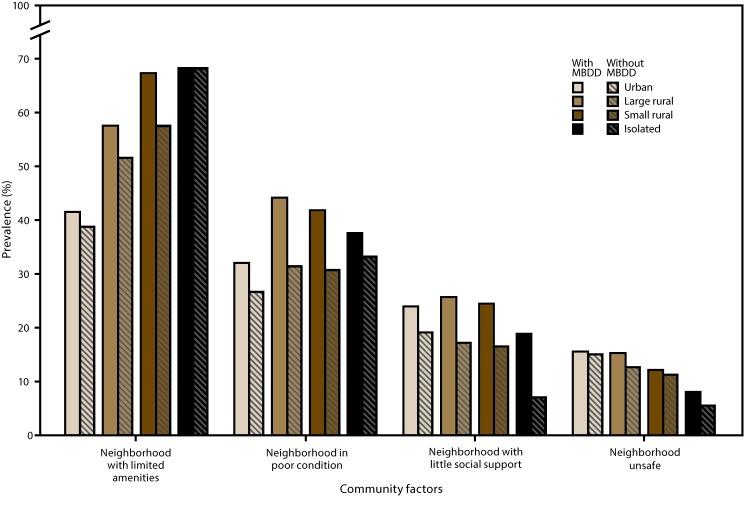
Prevalence of selected community factors* among children aged 2–8 years with and without mental, behavioral, and developmental disorders^†^ in urban and rural areas^§^ — National Survey of Children’s Health, United States, 2011–2012 **Abbreviations:** MBDD = mental, behavioral, or developmental disorder; RUCA = rural-urban commuting area. * **Neighborhood with limited amenities:** Based on responses of “no” to at least one of the following statements: “Please tell me if the following places and things are available to children in your neighborhood, even if [the child] does not actually use them”: 1) sidewalks or walking paths; 2) a park or playground area; 3) a recreation center, community center, or boys’ or girls’ club; 4) a library or bookmobile. **Neighborhood in poor condition:** Based on responses of “yes” to any of the following three questions: “In your neighborhood, is there litter or garbage on the street or sidewalk? How about poorly kept or rundown housing? How about vandalism such as broken windows or graffiti?” **Neighborhood with little social support:** Based on responses of “definitely agree,” “somewhat agree,” “somewhat disagree,” or “definitely disagree” to the following four statements about their neighborhood or community: “People in this neighborhood help each other out; we watch out for each other’s children in this neighborhood; there are people I can count on in this neighborhood; if my child were outside playing and got hurt or scared, there are adults nearby who I trust to help my child.” Responses were scored 1–4 (“definitely agree” through “definitely disagree”), and an average score was calculated; averages ≥2.25 indicated a lack of support. **Neighborhood unsafe:** Based on responses of “never” or “sometimes” (compared with “usually” or “always”) to the question, “How often do you feel [the child] is safe in your community or neighborhood?” ^†^ Significant differences in the prevalence of certain community factors were found between children with and without MBDDs in certain urban and rural areas. **Neighborhood with limited amenities:** no areas; **neighborhood in poor condition:** urban, large rural, and small rural areas; **neighborhood with little social support:** urban, large rural, and isolated areas; **neighborhood unsafe:** no areas. ^§^ Urban and rural designations were determined using the four-category classification of the 2006 RUCAs, a census tract–based classification system. Urban areas (RUCA codes 1.0, 1.1, 2.0, 2.1, 3.0, 4.1, 5.1, 7.1, 8.1, and 10.1) include metropolitan areas and surrounding towns from which commuters flow to an urban area; large rural areas (RUCA codes 4.0, 4.2, 5.0, 5.2, 6.0, and 6.1) include large towns (micropolitan areas) with populations of 10,000–49,999 and their surrounding areas; small rural areas (RUCA codes 7.0, 7.2, 7.3, 7.4, 8.0, 8.2, 8.3, 8.4, 9.0, 9.1, and 9.2) include small towns with populations of 2,500–9,999 and their surrounding areas; isolated areas (RUCA codes 10.0, 10.2, 10.3, 10.4, 10.5, and 10.6) are not near towns with a population of ≥2,500 (**Source:** US Department of Health and Human Services, Health Resources and Services Administration. The health and well-being of children in rural areas: a portrait of the nation 2011–2012. Rockville, MD: US Department of Health and Human Services; 2015. https://mchb.hrsa.gov/nsch/2011-12/rural-health/pdf/rh_2015_book.pdf).

Overall, a higher prevalence of children with an MBDD experienced health care and family challenges than children without an MBDD. Within urban areas only, children with an MBDD more often had inadequate health insurance than children without an MBDD. Children with an MBDD more often lacked a medical home in urban areas, small rural areas, and isolated areas than children without an MBDD. Regardless of urban or rural status, children with an MBDD more often than children without had at least one parent with fair or poor mental health. A higher percentage of parents of children with an MBDD reported financial difficulties within urban, large rural, and small rural areas (Figures 1 and 2); data for the figures are provided (https://stacks.cdc.gov/view/cdc/43792).

In urban, large rural, and small rural areas, children with an MBDD more often lived in a neighborhood in poor condition than children without an MBDD. Children with an MBDD in urban, large rural, and isolated rural areas lacked social support in their neighborhood more often than children in those types of areas who did not have an MBDD (Figures 1 and 2); data for the figures are provided (https://stacks.cdc.gov/view/cdc/43792).

Among children with an MBDD, a higher prevalence of those living in any rural area (large, small, and isolated combined) than those in urban areas had a parent with fair or poor mental health, lived in families with financial difficulties, lived in a neighborhood with limited amenities, and lived in a neighborhood in poor condition. After adjusting for race/ethnicity and poverty, the only factor that was no longer associated with rurality was financial difficulties (Table 3).

**TABLE 3:** Health care, family, and community factors among children aged 2–8 years with mental, behavioral, and developmental disorders in urban and rural areas — National Survey of Children’s Health, United States, 2011–2012

Variable	Urban*	Large rural, small rural, isolated*	Rural-urban prevalence ratio	Rural-urban adjusted prevalence ratio^†^
	% (95% CI)	% (95% CI)	(95% CI)	(95% CI)
**Health care**				
Inadequate insurance for optimal health	26.9 (24.2–29.8)	24.8 (20.7–29.5)	0.9 (0.8–1.1)	1.0 (0.8–1.2)
No medical home	57.4 (54.2–60.6)	52.4 (47.5–57.4)	0.9 (0.8–1.0)	0.9 (0.9–1.1)
**Family**				
At least one parent with fair or poor mental health	17.5 (14.9–20.5)	27.6 (22.3–33.6)	1.6 (1.2–2.0)^§^	1.3 (1.0–1.7)^¶^
Financial difficulties	33.9 (30.7–37.1)	41.5 (36.6–46.6)	1.2 (1.1–1.4)^¶^	1.0 (0.9–1.2)
**Community **				
Neighborhood with limited amenities	41.7 (38.5–45.0)	63.0 (57.9–67.7)	1.5 (1.4–1.7)^§^	1.5 (1.3–1.6)^§^
Neighborhood in poor condition	32.2 (29.1–35.4)	42.4 (37.5–47.5)	1.3 (1.1–1.5)^§^	1.2 (1.0–1.4)^¶^
Neighborhood with little social support	24.1 (21.5–27.0)	24.1 (20.1–28.7)	1.0 (0.8–1.2)	1.0 (0.8–1.2)
Neighborhood unsafe	15.7 (13.6–18.0)	13.0 (10.1–16.5)	0.8 (0.6–1.1)	0.9 (0.7–1.2)

**Abbreviations:** CI = confidence interval; FPL = federal poverty level; RUCA = rural-urban commuting area.

* Urban and rural designations were determined using the four-category classification of the 2006 RUCAs, a census tract–based classification system. Urban areas (RUCA codes 1.0, 1.1, 2.0, 2.1, 3.0, 4.1, 5.1, 7.1, 8.1, and 10.1) include metropolitan areas and surrounding towns from which commuters flow to an urban area; large rural areas (RUCA codes 4.0, 4.2, 5.0, 5.2, 6.0, and 6.1) include large towns (micropolitan areas) with populations of 10,000–49,999 and their surrounding areas; small rural areas (RUCA codes 7.0, 7.2, 7.3, 7.4, 8.0, 8.2, 8.3, 8.4, 9.0, 9.1, and 9.2) include small towns with populations of 2,500–9,999 and their surrounding areas; isolated areas (RUCA codes 10.0, 10.2, 10.3, 10.4, 10.5, and 10.6) are not near towns with a population of ≥2,500 (**Source:** US Department of Health and Human Services, Health Resources and Services Administration. The health and well-being of children in rural areas: a portrait of the nation 2011–2012. Rockville, MD: US Department of Health and Human Services; 2015. https://mchb.hrsa.gov/nsch/2011-12/rural-health/pdf/rh_2015_book.pdf).

^†^ Prevalence ratios adjusted for poverty (<200% FPL or ≥200% FPL) and race/ethnicity (non-Hispanic white or all other races/ethnicities).

^§^ Prevalence ratio significant at p<0.01.

^¶^ Prevalence ratio significant at p<0.05.

## Discussion

MBDDs are prevalent among young children. The findings in this report indicate that approximately one in six young children in rural communities had a diagnosed MBDD. A higher prevalence was found among children in small rural areas than among those in urban areas. Children in rural areas might live in neighborhoods with fewer resources (*14*) and also might experience more poverty-related factors and indicators of family adversity, such as lower parental education and poor parental mental health (*5*). Neighborhoods that provide access to community resources (e.g., playgrounds, libraries, and community centers) can promote school readiness and social development among young children (*15*). Within communities with few resources, having strong social connections with family, friends, and the neighborhood can offset some of the negative effects on parental mental health (e.g., stress and depression) if those connections involve positive models (*15*). In contrast, social isolation, which is common in rural areas, and poverty can place additional stress on parents, affecting their mental health and parenting behaviors (*19*), in turn affecting the health and development of their children. Factors such as poor housing conditions and living below the FPL were associated with increased psychological distress and allostatic load (a composite measure of physiologic stress responses) among a sample of school-aged children in rural New York counties (*20*). However, parenting behaviors that create healthy home environments and provide access to learning experiences in the home (e.g., reading to the child or having age-appropriate toys) and outside the immediate community (e.g., going to parks, libraries, or museums outside the neighborhood) have been shown to mediate child outcomes in neighborhoods with few resources (*15*).

These NSCH data and previous research indicate that children with MBDDs were more negatively affected by certain health care, family, and community factors than children without an MBDD (*2*). In addition, the data in this report demonstrate similar patterns of these differences across the rural-urban continuum, suggesting MBDD-related disparities exist regardless of residency type. However, among families of children with MBDDs, rural families had financial difficulties more often than urban families, and the children also more often had a parent with poor mental health and lived in neighborhoods lacking amenities and in poor condition. This suggests that parents of children in rural areas with MBDDs report more family and neighborhood adversity than parents of children in urban areas with MBDDs. Similarly, a previous study found that rural families caring for a child with special health care needs arrange and deliver more care in their home, have higher financial costs for health care, and have more financial difficulties associated with health care than urban families caring for a child with special health care needs (*13*).

Alternate, integrative models of care such as collaborations between providers of primary health care and behavioral health care, among school-based services, and between community and state agencies (e.g., cooperative extension and faith-based organizations) can improve access to behavioral health resources for children (*6*,*11*). Integrative models of primary care and behavioral health care can reduce health care costs and increase quality (*6*). Strategies that have been identified for improving access in rural areas also might be effective in urban areas, including cross-training of primary care and allied health professionals (*9*), school-based behavioral health services (*11*), and telemedicine (*6*, *9*). School-based behavioral health services have been associated with reduced stigma and transportation barriers (*11*). Although children in racial/ethnic minority groups might be less likely to use specialized mental health care in general than white children, in a study of rural North Carolina counties, school-based behavioral health services were most likely to be used for treatment and accessed equally among racial/ethnic groups (*12*). Telemedicine also holds substantial promise for improving access to behavioral health care; family-focused telemedicine and other telemedicine options for children are now more readily available (*14*,*19*). Parenting support programs, behavioral health care, and integrated community supports can help address access disparities, promote early intervention, and mitigate severity within rural communities (*6*).

Certain findings in this report were unexpected. For example, compared with young children in urban areas, a lower percentage of young children in isolated areas lacked a medical home, and a lower percentage had a parent with fair or poor mental health. This might suggest that the previously mentioned community social support mitigated these factors. Having a medical home improves access to behavioral health services and improves family functioning and school participation among children with special health care needs in rural areas (*21*). In addition, children in rural communities, overall and by category, did not have different levels of insurance coverage than those in urban communities. Only within urban communities did a higher percentage of children with MBDDs lack health insurance than children without MBDDs. Other research indicates that children in rural areas are more likely than children in urban areas to have public health insurance (*5*). This suggests that having a child with an MBDD might be associated with unique health care factors for families in urban communities. Collaboration among health care, family, and community services and systems can address insufficient access to services (*21*) and promote the health and development of young children with MBDDs both in rural and urban communities (*2*,*9*).

Experiences among children in rural areas can vary substantially, and young children in certain rural communities might lack family and neighborhood resources more than children in other rural communities. Isolated rural and small rural areas also might offer more neighborhood social support than other rural communities. Focusing on the strengths of the close relationships among some rural families through family-focused care is an approach that might help address the mental health needs both of parents and children (*19*).

## Limitations

The findings in this report are subject to several limitations. First, relying on parent report of MBDD diagnosis by a health care provider is subject to recall error and potential social desirability biases and does not include children with undiagnosed MBDDs. Research indicates that residents in rural areas might underreport mental health disorders (*6*); therefore, the findings might not represent the association between MBDDs and rural residency. Second, the data are cross-sectional, and direction of effects or inferences about causality cannot be made. Third, urban and rural communities might define and conceptualize neighborhoods in different ways; therefore, the responses to these questions might differ in ways not fully measured by the questions administered. Fourth, the coding variable used to define rurality describes rurality/urbanicity by population density and work commuting patterns. These codes are based on 2000 census data and 2004 zip codes; designations of urban areas can change over time. Fifth, because these are cross-sectional data and represent a single point in time, they do not reflect changes in residence (e.g., the possibility that a child moved from an urban area to a rural area or the converse). Sixth, previous research indicates that rurality is significantly associated with poverty and other demographic factors (*5*); as such, the individual contributions of factors in this report might be difficult to discern. Finally, these data might be affected by nonresponse bias even though they have been weighted to adjust for demographic biases that might have resulted from the low response rate.

## Future Directions

Research examining neighborhood risk and protection for childhood mental health disorders within rural communities is sparse. Longitudinal studies of MBDDs, rurality, and sociodemographic, health care, and community factors could provide additional data regarding long-term outcomes, direction of effects, and trends over time. In addition, examining these health care, family, and community factors and their associations with specific MBDDs (e.g., ADHD or speech and language problems) by rural area would allow for better understanding of barriers and facilitators that could be used to develop specific approaches to improving the diagnosis and treatment of these disorders in different locations. Additional analyses could explore associations between MBDDs and other health care variables, such as the receipt of mental health treatment or the number of health care visits in the past year. In addition, analyses examining specific disorders could help communities identify specific strengths and opportunities.

## Conclusion

Children in rural communities more often experience poverty and live in communities that are lacking in amenities and are in poor condition; these factors have been previously associated with MBDDs among young children (*2*,*15*). Research also indicates that factors such as access to medical services, resource-seeking behaviors among parents, and community social connections might mitigate some of the negative health and developmental effects of living in higher poverty neighborhoods (*15*). MBDDs are prevalent among young children in various rural and urban communities; many health care, family, and community disparities were reported between children with and without MBDDS within rural and urban categories. Integrative models of behavioral and physical health care can help promote the health and development of young children (*2*) and might address some of the unique barriers experienced by children living in rural communities (*6*,*11*). Collaboration among and within early learning and parenting support programs, health care systems, and economic systems can help promote the health and development of young children in rural communities by facilitating family access to behavioral health care and community social and recreational resources. Addressing rural-urban disparities in neighborhood resources that allow children to play, read, and socialize also might present opportunities for prevention and treatment.

## References

[1] US Department of Health and Human Services, Office of Disease Prevention and Health Promotion. Healthy People 2020 [Internet]. Washington, DC: US Department of Health and Human Services. https://www.healthypeople.gov/node/3498/objectives#4816

[2] Bitsko RH, Holbrook JR, Robinson LR, ; EdS. EdS. Health care, family, and community factors associated with mental, behavioral, and developmental disorders in early childhood—United States, 2011–2012. MMWR Morb Mortal Wkly Rep 2016;65:221–6. 10.15585/mmwr.mm6509a1 26963052

[3] Meit M, Knudson A, Gilbert T, The 2014 update of the rural-urban chartbook. Rural Health Reform Policy Research Center. Bethesda, MD 2014.

[4] Howell E, McFeeters J. Children’s mental health care: differences by race/ethnicity in urban/rural areas. J Health Care Poor Underserved 2008;19:237–47. 10.1353/hpu.2008.0008 18263999

[5] Anderson NJ, Neuwirth SJ, Lenardson JD, Hartley D. Patterns of care for rural and urban children with mental health problems. Portland, ME: Maine Rural Health Research Center; 2013. http://muskie.usm.maine.edu/Publications/MRHRC/WP49-Rural-Children-Mental-Health.pdf

[6] Gamm L, Stone S, Pittman S. Mental health and mental disorders—a rural challenge: a literature review. Rural Healthy People 2010: a companion document to Healthy People 2010. Vol. 2. College Station, TX; 2003:97–113. https://sph.tamhsc.edu/srhrc/docs/rhp-2010-volume2.pdf

[7] Heflinger CA, Shaw V, Higa-McMillan C, Lunn L, Brannan AM. Patterns of child mental health service delivery in a public system: rural children and the role of rural residence. J Behav Health Serv Res 2015;42:292–309. 10.1007/s11414-015-9464-9 25813915

[8] Health Resources and Services Administration. Mental health and rural America: 1994–2005. Rockville, MD: US Department of Health and Human Services, Health Resources and Services Administration, Office of Rural Health Policy; 2006. https://www.ruralhealthresearch.org/mirror/6/657/RuralMentalHealth.pdf

[9] Institute of Medicine. Quality through collaboration: the future of rural health. Washington, DC: National Academies Press; 2005. https://www.nap.edu/read/11140/chapter/1

[10] Health Resources and Services Administration. Designated health professional shortage areas statistics. Rockville, Maryland: US Department of Health and Human Services, Health Resources and Services Administration; 2016.

[11] Smalley KB, Yancey CT, Warren JC, Naufel K, Ryan R, Pugh JL. Rural mental health and psychological treatment: a review for practitioners. J Clin Psychol 2010;66:479–89. 20222125 10.1002/jclp.20688

[12] Angold A, Erkanli A, Farmer EM, Psychiatric disorder, impairment, and service use in rural African American and white youth. Arch Gen Psychiatry 2002;59:893–901. 10.1001/archpsyc.59.10.893 12365876

[13] Skinner AC, Slifkin RT. Rural/urban differences in barriers to and burden of care for children with special health care needs. J Rural Health 2007;23:150–7. 10.1111/j.1748-0361.2007.00082.x 17397371

[14] Council of Economic Advisers Domestic Policy Council and Office of Management and Budget. Opportunity for all: fighting rural child poverty. Washington, DC: White House; 2015. http://sustainableagriculture.net/blog/rural-child-poverty/

[15] Leventhal T, Brooks-Gunn J. The neighborhoods they live in: the effects of neighborhood residence on child and adolescent outcomes. Psychol Bull 2000;126:309–37. 10.1037/0033-2909.126.2.309 10748645

[16] Health Resources and Services Administration. The health and well-being of children in rural areas: a portrait of the nation 2011–2012, The National Survey of Children’s Health 2015. Rockville, Maryland: US Department of Health and Human Services, Health Resources and Services Administration; 2015.

[17] CDC, National Center for Health Statistics. State and local area integrated telephone survey. 2011–2012 National Survey of Children’s Health frequently asked questions. Rockville, MD: US Department of Health and Human Services, CDC, National Center for Health Statistics; 2013. https://www.cdc.gov/nchs/slaits/nsch.htm

[18] Washington, Wyoming, Alaska, Montana, Idaho (WWAMI) Rural Health Research Center, US Department of Agriculture, Economic Research Service. Rural-urban commuting area codes. Seattle, WA. http://depts.washington.edu/uwruca

[19] Heflinger CA, Christens B. Rural behavioral health services for children and adolescents: an ecological and community psychology analysis. J Community Psychol 2006;34:379–400. 10.1002/jcop.20105

[20] Evans GW. A multimethodological analysis of cumulative risk and allostatic load among rural children. Dev Psychol 2003;39:924–33. 10.1037/0012-1649.39.5.924 12952404

[21] Farmer JE, Clark MJ, Sherman A, Marien WE, Selva TJ. Comprehensive primary care for children with special health care needs in rural areas. Pediatrics 2005;116:649–56. 10.1542/peds.2004-0647 16140704

